# Falciform ligament abscess secondary to a ruptured liver abscess in a child: a case report

**DOI:** 10.11604/pamj.2020.35.21.8592

**Published:** 2020-01-24

**Authors:** Abdelhalim Mahmoudi, Mohammed Rami, Khalid Khattala, Aziz El Madi, Youssef Bouabdallah

**Affiliations:** 1 Department of Pediatric Surgery, CHU Hassan II, Faculty of Medicine and Pharmacy, Sidi Mohamed Ben Abdellah University, Fez, Morocco

**Keywords:** Falciform ligament, abscess, acute abdomen, liver abscess

## Abstract

Abscess of the liver ligaments is extremely rare, and abscess of the falciform ligament has been sporadically reported. We report the case of a 3 years old male who presented with a three days history of right upper quadrant abdominal pain, fever and nausea. The ultrasound and computed tomography (CT) scan showed an abdominal wall abscess located anterior to the liver. The patient underwent surgery. Abscess of the falciform ligament secondary to a ruptured liver abscess was found. Excision of the falciform ligament including the abscess was performed. Although pathology of the falciform ligament is rare, it should be included in the differential diagnosis of acute abdomen.

## Introduction

Few cases of falciform ligament abscess have been reported. This implies that the pathology of falciform ligament abscess is poorly understood, and many surgeons may be unable to recognize it when encountered. We report a case of a pyogenic falciform ligament abscess secondary to a ruptured liver abscess.

## Patient and observation

**Figure 1:**
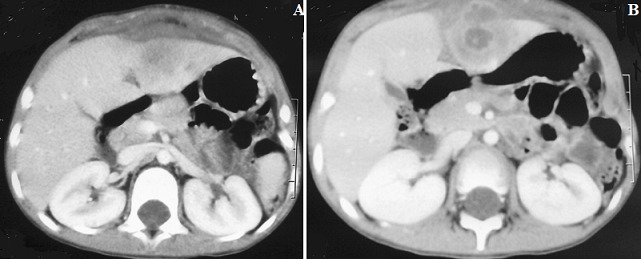
A) CT scan showing abscess involving in the left lobe of liver with extension into the overlying anterior abdominal wall; B) the early phase of the contrast-enhanced CT scan revealed a strongly enhanced margin of the mass liver and no enhancement of the inside of the mass

**Figure 2:**
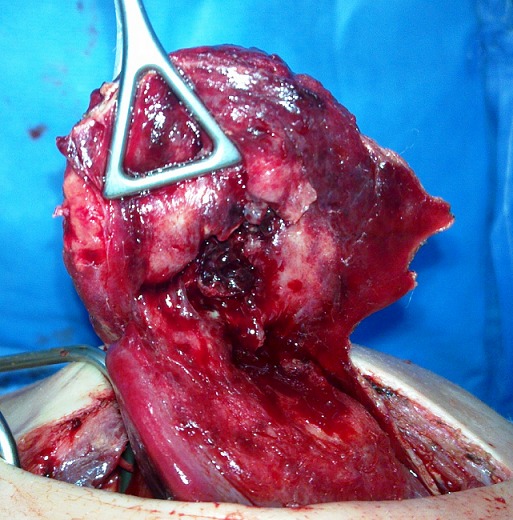
Operative photograph: falciform ligament abscess communicating with liver abscess

A 3-year-old male was admitted to the hospital with a three-day history of right upper quadrant pain, fever, diarrhea and nausea. There was no history of jaundice. There was no history of abdominal surgery or abdominal trauma. On physical examination, her body temperature was 38.2°C, pulse was 84 beats/min and blood pressure was 110/70 mmHg. Abdominal examination revealed a palpable epigastric mass that was slightly tender. There were no signs of peritonitis. Results of laboratory studies showed: white blood cell count 19,500/ml with 75.0% polymorphonuclear leukocytes, c-reactive protein test (CRP) was 130 mg/dl. Liver function and electrolytes were within normal ranges. Abdominal radiography was unremarkable. An ultrasound examination revealed an anechoic lesion measuring 5×4 cm in the left lobe of the liver. The biliary ducts, gallbladder, pancreas and spleen were normal (Figure 1 A, Figure 1 B). A provisional diagnosis of liver abscess was made. Subsequently a CT scan revealed a mass measuring 4 x 3 cm, and arising from the left lobe of the liver with surrounding inflammatory changes and extension into the overlying anterior abdominal wall (Figure 2) with a strongly enhanced margin of the mass and no enhancement of the inside of the mass. Surgical exploration confirmed an abscess in the falciform ligament communicating with rupture of a liver abscess. The abdominal wall was clear. The abscess was drained and cultures were obtained. The falciform ligament was resected. Intraoperative cultures grew *S. aureus* (methicillin sensitive). Antibiotics were appropriately manipulated, and he was kept on oral antibiotic therapy for 4 weeks. Echocardiography revealed no evidence of valvular heart disease. The patient was discharged on postoperative day 7. Pathological examination revealed a fibrosis of the falciform ligament with abscess formation, and presence of non-specific granulomatous tissue. The post-operative course was uneventful.

## Discussion

The falciform ligament is the embryologic remnant of the ventral mesentery, and marks the separation of the most caudal part of the left lobe of the liver into medial and lateral segments. The ligament is composed of two mesothelial layers, within which lies the ligamentum teres hepatis (obliterated left umbilical vein), paraumbilical veins, muscular fibers, and a variable amount of adipose tissue [1]. Falciform ligament abscess is a rare clinical entity, with only a few reports noted in the literature [2]. Infections can extend from the liver, gallbladder [3, 4] and umbilicus [5]. An infection of a cystic lesion of the falciform ligament has been reported as a cause of a falciform ligament abscess [6]. As shown in this case, it is important to suspect a falciform ligament abscess in a patient with a right upper quadrant abscess and a prior history of abdominal infections. The presence of right upper quadrant abdominal pain, epigastric tenderness, fever, leukocytosis, and a mass in the anterior abdomen should raise suspicion of falciform ligament abscess [2, 4, 7]. Ultrasound and computed tomography scans should be helpful in detecting the presence of an abscess. On computed tomography scanning, free air limited to the area surrounding the falciform ligament indicates the presence of an abscess [1]. Previous authors reported successful treatment of the falciform ligament abscess after excision of the ligament [5, 7, 8]. Therefore, when a falciform ligament abscess is suspected, surgical excision rather than percutaneous drainage should be considered for the initial treatment. Depending on surgeon’s expertise, patient’s condition, and severity and extent of disease either open or laparoscopic surgery may be performed. Rupture abscess liver is considered the cause of the falciform ligament abscess presented here. Number of pediatric cases of pyogenic liver abscess is limited. It usually results from seeding of the liver by pathogenic bacteria via hematogenous route. The most common etiologic agent is *Staphylococcus aureus*, and most often, a solitary abscess is found such as our patient, if bacteria spread from an adjacent infected organ, the abscess is usually multiple and polymicrobial, with gram-negative enterics and anaerobes.

## Conclusion

Infection of the falciform ligament is extremely rare, but it should be suspected in patient with abdominal complaints and imaging study may be useful to demonstrate abscess formation of the falciform ligament.
